# Reproducibility and repeatability of central corneal thickness measurement in healthy eyes using four different optical devices

**DOI:** 10.1007/s10792-016-0369-2

**Published:** 2016-10-08

**Authors:** Remzi Karadag, Murat Unluzeybek, Ozgur Cakici, Ayse Yagmur Kanra, Huseyin Bayramlar

**Affiliations:** 1 Department of Ophthalmology, Istanbul Medeniyet University School of Medicine, Goztepe, Istanbul, Turkey; 2 Department of Ophthalmology, Istanbul Medeniyet University Goztepe Research and Training Hospital, Goztepe, Istanbul, Turkey

**Keywords:** Central corneal thickness, Pachymetry, Optical low-coherence reflectometry, Optic coherence tomography, Specular microscopy, Corneal topography

## Abstract

**Aim:**

The aim of the study is to compare the measurements of central corneal thickness (CCT) performed by two examiners with four different methods at different times inter- and intra-individually.

**Methods:**

Thirty healthy people were included in the study. In these measurements, an optical low-coherence reflectometry (OLCR), an optic coherence tomography (OCT), a specular microscopy (SM), and a corneal topography (CT) were used. Two examiners performed the measurements in a consecutive manner. After 1–7 days of the first measurements, the second measurements were performed again consecutively. The mean of three measurements was taken in each session for all devices.

**Results:**

In OCT measurements, there was a significant difference between two examiners in both sessions (*p* < 0.001), while no significant differences were found between two examiners in first and second sessions in SM, CT, and OLCR measurements. When each examiner’s measurements were compared to two sessions, there were no significant differences (*p* > 0.05, for all) except the SM measurements of the first examiner (*p* = 0.041). When the first measurements of two examiners were compared, the smallest values were of OCT. At the first session of two examiners, there was a significant difference between OCT and CT measurements, and between OCT and OLCR (*p* < 0.001, *p* = 0.002 for the first examiner and *p* < 0.001 for the second examiner, respectively).

**Conclusion:**

Our study showed that CCT measurements made by CT and OLCR methods were almost same and highly correlated for both the examiners’ measurements. CCTs measured by OCT were on average 30 μm thinner than CT and OLCR.

## Introduction

Central corneal thickness (CCT) has a great deal of importance in the diagnosis and follow-up of glaucoma and refractive surgical interventions such as cross-linking [1]. Furthermore, measurement of CCT is one of the indirect methods to evaluate whether the cornea endothelium is healthy or not. Therefore, CCT measurement, which can simply be performed through various methods, is also used in the follow-up of some diseases such as endothelial dystrophies [2]. Ultrasonic Pachymeter is the gold standard. In this method, however, there are some limitations, such as direct contact of the equipment with the cornea, measurement of the estimated central point, limited definition of the thinnest point of the cornea, and variation according to the individual performing the measurement. Currently, devices such as optical, low-coherence reflectometry, optical coherence tomography, noncontact specular microscopy, and corneal topography have been developed to measure CCT with a noncontact optic method [3]. These noncontact methods are less dependent on the operator and have the advantage of demonstrating the thinnest parts of the cornea. Thus, the reliability of the CCT measurements is very important in the diagnosis and management of various corneal diseases [2].

The aim of this study is to compare the measurements and operating individuals in terms of measurement values obtained in four different noncontact methods of CCT measurement performed by two different individuals, to compare the measurements they perform in different time periods, and to evaluate whether these methods can be interchangeable or not.

## Materials and methods

Thirty individuals who applied to our clinic were included in the study. All cases were informed about the study, and informed consent was obtained. This study was performed in accordance with the Helsinki Declaration criteria. Thirty right eyes of 14 male and 16 female patients without any systemic diseases and aged between 22 and 60 years were included in the study. Patients with prior ocular surgery, the presence of glaucoma, refractive errors greater than ±2 diopters, cataracts, corneal opacity, and keratoconus were excluded from the study.

Optical low-coherence reflectometry (OLCR; LenStar LS900; Haag-Streit AG, Koeniz, Switzerland), optical coherence tomography (OCT; Topcon 3D OCT-2000, Topcon Medical System, Oakland, New Jersey, USA), noncontact Specular Microscopy (SM; Topcon SP-3000P; Topcon Corporation, Tokyo, Japan), and corneal topography (CT; Sirius; Costruzione Strumenti Oftalmici, Florence, Italy) equipments were used to measure CCT.

Corneal thickness measurements were performed by two individuals consecutively. One to seven days after the first measurement, second measurements were performed in all patients by the same two examiners. Measurements were performed between 10 a.m. and 2 p.m. to avoid diurnal variations. Three measurements were performed on each piece of equipment, and the means were calculated.

SPSS 16 program was used in the statistical analysis, and parametric tests that were used for the distribution of the data were normal. The level of significance was accepted as *p* < 0.05.

## Results

Fourteen males and 16 females, with a mean age of 30.97 ± 7.75 years, were included in the study. The results of the first and second measurements performed by the first and second investigators are shown in Table 1. A statistically significant correlation was present between the first and second measurements conducted by both investigators in all equipments (in all: *p* < 0.001). A powerful correlation was found between the measurements of the two sessions in the SM, CT, and OLCR methods, while a correlation of an intermediate level was present between the measurements performed using OCT equipment (*r*: 0.747, *r*: 0.691, Table 1, respectively).

**Table 1:** Correlation between researchers of both the first and second measurements in the same device (*r*: Pearson correlation coefficient)

Methods	First session (μm)	Second session (μm)	*r* value	*p* value
First examiner				
OCT	514.18 ± 25.60	512.60 ± 20.85	0.747	<0.001
Specular microscopy	525.54 ± 34.64	523.21 ± 34.51	0.985	<0.001
Corneal topography	547.46 ± 32.96	546.61 ± 31.44	0.989	<0.001
OLCR	544.25 ± 31.16	544.64 ± 30.90	0.987	<0.001
Second examiner				
OCT	502.06 ± 22.33	499.24 ± 22.29	0.691	<0.001
Specular microscopy	524.53 ± 34.60	524.13 ± 33.81	0.988	<0.001
Corneal topography	546.69 ± 32.14	546.12 ± 31.22	0.989	<0.001
OLCR	544.81 ± 30.96	544.45 ± 32.49	0.986	<0.001

*OCT* optic coherence tomography, *OLCR* optical low-coherence reflectometry

In terms of the measurements of the same individual in both sessions, no significant differences were found between any first and second session measurements of the same individual (*p* > 0.05 for all, Table 2), except the SM measurements of the first investigator (*p* = 0.041).

**Table 2:** Statistical differences between researchers of both the first and second measurement values of the same unit

Devices	First session mean (range)	Second session mean (range)	*p* value
First examiner (μm)			
OCT	514.18 ± 25.60 (455.67–553.00)	512.60 ± 20.85 (469.00–548.00)	0.617
Specular microscopy	525.54 ± 34.64 (462.33–579.00)	523.21 ± 34.51 (460.00–573.00)	**0.041**
Corneal topography	547.46 ± 32.96 (497.33–602.33)	546.61 ± 31.44 (492.00–600.00)	0.355
OLCR	544.25 ± 31.16 (494.33–594.00)	544.64 ± 30.90 (491.67–597.33)	0.672
Second examiner			
OCT	502.06 ± 22.33 (459.33–546.67)	499.24 ± 22.29 (460.67–551.33)	0.387
Specular microscopy	524.53 ± 34.60 (466.00–581.00)	524.13 ± 33.81 (456.67–573.67)	0.689
Corneal topography	546.69 ± 32.14 (492.33–597.33)	546.12 ± 31.22 (494.67–593.67)	0.521
OLCR	544.81 ± 30.96 (494.00–598.00)	544.45 ± 32.49 (486.33–592.00)	0.716
Differences between two examiners			
OCT	14.37 ± 9.52 (0.33–35.00)	16.87 ± 14.16 (1.66–58.00)	0.759
Specular microscopy	3.86 ± 4.30 (0.00–20.00)	3.30 ± 2.52 (0.33–10.34)	0.474
Corneal topography	2.81 ± 2.35 (0.00–9.34)	2.60 ± 1.99 (0.00–6.66)	0.725
OLCR	2.80 ± 2.29 (0.00–9.00)	3.58 ± 3.59 (0.00–16.66)	0.933

Bold values are statistically significant measurements

*OCT* optic coherence tomography, *OLCR* optical low-coherence reflectometry

When the two investigators were compared with each other based on the measurements obtained, the measurements by both investigators using SM, CT, and OLCR were similar (in all: *p* > 0.05); however, OCT measurements by the two investigators were different from each other, both in the first (*p* < 0.001) and second sessions (*p* < 0.001) (Table 3). When the first and second session measurements performed using different types of equipment were compared to each other, the difference between the two sessions was statistically significant in the OCT measurements, but no difference was found in the two measurements in other equipment (Table 3). SM, CT, and OLCR measurements demonstrated a powerful correlation between the two investigators in both sessions. OCT measurements by the two investigators were highly correlated in the first session (*r*: 0.876) and intermediately correlated in the second session (*r*: 0.668) (Table 4).

**Table 3:** Statistical differences between the two session’s measurement values of the examiners in the same method

Methods	First examiner mean (range)	Second examiner mean (range)	*p* value
First session (μm)			
OCT	514.18 ± 25.60 (455.67–553.00)	502.06 ± 22.33 (459.33–546.67)	**<0.001**
Specular microscopy	525.54 ± 34.64 (462.33–579.00)	524.53 ± 34.60 (466.00–581.00)	0.341
Corneal topography	547.46 ± 32.96 (497.33–602.33)	546.69 ± 32.14 (492.33–597.33)	0.255
OLCR	544.25 ± 31.16 (494.33–594.00)	544.81 ± 30.96 (494.00–598.00)	0.405
Second session (μm)			
OCT	512.60 ± 20.85 (469.00–548.00)	499.24 ± 22.29 (460.67–551.33)	**<0.001**
Specular microscopy	523.21 ± 34.51 (460.00–573.00)	524.13 ± 33.81 (456.67–573.67)	0.227
Corneal topography	546.61 ± 31.44 (492.00–600.00)	546.12 ± 31.22 (494.67–593.67)	0.419
OLCR	544.64 ± 30.90 (491.67–597.33)	544.45 ± 32.49 (486.33–592.00)	0.832
Differences between two seasons			
OCT	13.15 ± 10.80 (0.00–38.00)	12.32 ± 12.61 (0.00–51.33)	0.349
Specular microscopy	4.71 ± 4.29 (1.00–19.67)	3.93 ± 3.66 (0.00–16.66)	0.531
Corneal topography	3.89 ± 3.01 (0.00–12.33)	3.63 ± 3.01 (0.33–15.34)	0.721
OLCR	3.85 ± 3.13 (0.34–12.33)	3.90 ± 3.76 (0.33–17.34)	0.302

Bold values are statistically significant measurements

*OCT* optic coherence tomography, *OLCR* optical low-coherence reflectometry

**Table 4:** Correlation between both session to the values of the researchers in the same device (*r*: Pearson correlation coefficient)

Methods	First examiner	Second examiner	*r* value	*p* value
First session (μm)				
OCT	514.18 ± 25.60	502.06 ± 22.33	0.876	<0.001
Specular microscopy	525.54 ± 34.64	524.53 ± 34.60	0.986	<0.001
Corneal topography	547.46 ± 32.96	546.69 ± 32.14	0.994	<0.001
OLCR	544.25 ± 31.16	544.81 ± 30.96	0.993	<0.001
Second session (μm)				
OCT	512.60 ± 20.85	499.24 ± 22.29	0.668	<0.001
Specular microscopy	523.21 ± 34.51	524.13 ± 33.81	0.993	<0.001
Corneal topography	546.61 ± 31.44	546.12 ± 31.22	0.995	<0.001
OLCR	544.64 ± 30.90	544.45 ± 32.49	0.988	<0.001

*OCT* optic coherence tomography, *OLCR* optical low-coherence reflectometry

A significant difference was also found between the OCT measurements and CT and OLCR measurements of the first session by both investigators (for the measurements of the first investigator: *p* < 0.001, *p* = 0.002 and for the measurements of second investigator *p* < 0.001, *p* < 0.001, respectively).

The measurements of the first investigator using OCT and SM equipment demonstrated no difference (*p* = 0.972), except for between the measurements of the second investigator (*p* = 0.029), (Table 5).

**Table 5:** A comparison (P values) of the first session of the researchers measurements (Bonferroni multiple comparison)

Devices		OCT	Specular microscopy	Corneal topography	OLCR
First examiner					
OCT (514.18 ± 25.60 µm)			0.972	<0.001	0.002
Specular microscopy (525.54 ± 34.64 µm)		0.972		0.046	0.134
Corneal topography (547.46 ± 32.96 µm)		<0.001	0.046		1.000
OLCR (544.25 ± 31.16 µm)		0.002	0.134	1.000	
Second examiner					
OCT (502.06 ± 22.33 µm)			0.029	<0.001	<0.001
Specular microscopy (524.53 ± 34.60 µm)		0.029		0.033	0.066
Corneal topography (546.69 ± 32.14 µm)		<0.001	0.033		1.000
OLCR (544.81 ± 30.96 µm)		<0.001	0.066	1.000	

*OCT* optic coherence tomography, *OLCR* optical low-coherence reflectometry

When the SM and CT measurements by both investigators were compared, significant differences were found between the measurements (*p* = 0.046 and *p* = 0.033 for the first and second investigators, respectively (Table 5). No significant difference was found between the SM and OLCR measurements (*p* = 0.134 for the first investigator, *p* = 0.066 for the second investigator) (Table 5). No significant differences were also found between the CT and OLCR measurements by both investigators (*p* = 1.0) (Table 5).

When the correlations of the results of the different methods were analyzed, the first measurements by both investigators were statistically significant (in all: *p* < 0.001) (Table 6).

**Table 6:** The researchers compared in each of the first measurements of all devices (r: Pearson Correlation Coefficient)

Devices	OCT		Specular microscopy		Corneal topography		OLCR	
	*r*	*p*	*r*	*p*	*r*	*p*	*r*	*p*
First researcher								
OCT			0.655	<0.001	0.627	<0.001	0.644	<0.001
Specular microscopy	0.655	<0.001			0.966	<0.001	0.974	<0.001
Corneal topography	0.627	<0.001	0.966	<0.001			0.984	<0.001
OLCR	0.644	<0.001	0.974	<0.001	0.984	<0.001		
Second researcher								
OCT			0.654	<0.001	0.678	<0.001	0.632	<0.001
Specular microscopy	0.654	<0.001			0.976	<0.001	0.984	<0.001
Corneal topography	0.678	<0.001	0.976	<0.001			0.980	<0.001
OLCR	0.632	<0.001	0.984	<0.001	0.980	<0.001		

*OCT* optic coherence tomography, *OLCR* optical low-coherence reflectometry

The results of SM, CT, and OLCR measurements were highly correlated with each other, while the results of OCT were intermediately correlated with the other three methods (Table 6).

## Discussion

Until recently, the single method used in the measurement of corneal thickness was an ultrasound-based method. However, this method has some disadvantages such as relatively variable results due to the difficulties in centralization and accommodation, the necessity for topical anesthesia, the possibility of epithelial damage due to contact between the probe and the cornea, and the risk of infection [4, 5]. CCT was reported to show a ±10-μm variation following topical anesthesia application [6, 7].

Advances in the diagnostic technologies and increased interest in corneal refractive surgical techniques resulted in the development of less invasive and noncontact methods for the measurement of the corneal thickness [8]. Since CCT measurements might be performed using many types of noncontact equipment, different types of equipments are available in different clinics. For the follow-up of the patients, a correlation of the results of the measurements performed by the equipment among each other and within a single piece of equipment is still important.

In the study of Borrego-Sanz L et al. which included 76 healthy patients using Pentacam, Ultrasound Pachymetry, Specular Microscopy, and Optic Biometer Lenstar LS 900 equipment, the CCT measurement results were reported as 565.36 ± 36.34, 549.61 ± 32.64, 537.24 ± 36.34, and 443.06 ± 34.28 μm, respectively. They found the highest correlation between Lenstar and UP [18]. Similarly, Tai et al. also found a high correlation between Lenstar and UP in their study that was performed on 184 eyes [9]. O’Donnell et al. [10] and Zhao et al. [11] found a high correlation between CCT measurements performed by Pentacam and Lenstar; however, they reported that these two devices could not be used in place of each other in clinical applications. Although a good correlation has been reported in UP and Pentacam in many studies, Tai et al. [19] found that Pentacam values were 10 μm higher than the UP values. They suggested such a difference may have originated from the fact that lacrima is also measured during measurement by Pentacam, and that UP applies pressure on the cornea. Moreover, Almubrad et al. [6] reported that CCT values measured by SM were lower than the values of UP (28.17 ± 19.20 μm), and they concluded that the two methods could not be used in place of each other.

Huang et al. [12] measured CCT in 66 healthy corneas and reported the results as follows: 538.82 ± 26.46 μm with Pentacam, 542.14 ± 27.12 μm with Sirius, 548.10 ± 26.41 μm with Galilei, and 532.81 ± 26.24 μm with RTVue. Repeatability and reproducibility were very high in these four devices, and it was suggested to be adequate for clinical use. Although the measurements of these devices were not correlated with each other since they were different, they emphasized that Galilei yielded better results. Additionally, using similar optic methods (Pentacam and RTVue), Nam et al. [13] and Chen et al. [14] achieved high intraobserver repeatability and interobserver reproducibility.

Since there are no studies of intra- and interobserver comparisons using the equipment we used in this study, it was not possible to perform a one-to-one comparison with other studies. The present study revealed that interobserver repeatability of OCT was poor, and the differences between the measurements were significant. Interobserver repeatability of other methods was good. We found interobserver and intraobserver repeatability of the measurements performed by OLCR and CT devices, and this value was less than 1 µm.

Milla et al. [15] and Savini et al. [16] found high repeatability in CCT measurements performed by Sirius and Galilei in the same order. Also, Khoramnia et al. [17] and Milla et al. [15] reported good repeatability in the same order using Pentacam and Sirius. Savini et al. [18] found the repeatability of Sirius slightly poorer than Galilei and slightly better than Pentacam. Many investigators [19, 20] found CCT measurement repeatability of Pentacam to be poorer compared to FD-OCT. A probable cause of this was reported to be the more rapid screening capacity of OCT.

In the present study, the results of measurements by OCT were lower than the values obtained with other equipment. Furthermore, while OCT measurement results were intermediate–high correlated with the results of other equipment, the results of other equipment were highly correlated with each other.

Current and some previous studies have suggested that different devices for CCT evaluation cannot be used in place of each other [21–23]. However, there are also studies reporting correlations from intermediate to excellent levels between Galilei and Orbscan II, and between Pentacam and Orbscan II [16, 24, 25]. In one study, the results of Orbscan II were markedly lower than the measurements performed using Pentacam and Galilei [26]. On the contrary, Sedaghat et al. [27] found the CCT values measured with Orbscan II to be slightly higher than the values obtained using Pentacam. Bueh et al. found no significant differences between the measurements of these two pieces of equipment [28]. However, in another study by Amano et al., no significant difference was found between the measurements of Orbscan II and Pentacam [29]. Kim et al. found that the CCT measurements prior to photorefractive keratectomy by Orbscan II in myopic patients were similar with the results of Pentacam; however, they reported that postoperative early and late measurements by Orbscan II resulted in thinner values [30].

In conclusion, values obtained in the present study by CT and OLCR methods taken by different individuals and the measurements obtained by the same individuals were almost similar and highly correlated. The difference between the results of these two measurement methods was approximately 1 µm. Since a significant difference was present in the results of the measurements of two different individuals performed by OCT, the reliability of this method in interobserver measurements in the evaluation of CCT is lower compared to the other methods according to our study. In addition, OCT measurements yield the lowest values compared to the other methods. We consider that the presence of a difference of at least 30 μm between the measurement values of CT and OCLR, and measurements of OCT will affect the calculations for corrected intraocular pressure and plan the operations in refractive surgery.
